# Seasonal Coronavirus‐Induced Immunological Imprinting and Previous Herpesvirus Infections in Patients With Long COVID

**DOI:** 10.1002/jmv.70582

**Published:** 2025-08-30

**Authors:** W. Ashwin Mak, Daphne Wapperom, Anne‐Lotte Redel, Johannes G. M. Koeleman, Pieter W. Smit, Wai‐Kwan Lam‐Tse, Tom van der Poll, Hung‐Jen Chen, Jeroen den Dunnen, Gert‐Jan Braunstahl, David S. Y. Ong

**Affiliations:** ^1^ Department of Medical Microbiology and Infection Control Franciscus Hospital Rotterdam the Netherlands; ^2^ Center for Infection and Molecular Medicine, Amsterdam Institute for Immunology and Infectious Diseases Amsterdam University Medical Centers Amsterdam the Netherlands; ^3^ Department of Pulmonology Franciscus Hospital Rotterdam the Netherlands; ^4^ Department of Respiratory Medicine Erasmus MC, University Medical Center Rotterdam Rotterdam the Netherlands; ^5^ Department of Medical Microbiology Molecular Diagnostics Unit, Maasstad Hospital Rotterdam the Netherlands; ^6^ Department of Rheumatology and Clinical Immunology Franciscus Hospital Rotterdam the Netherlands; ^7^ Department of Epidemiology Julius Center for Health Sciences and Primary Care, University Medical Center Utrecht Utrecht the Netherlands

**Keywords:** antibodies, autoimmunity, coronaviridae, cytomegalovirus, herpesviridae, long COVID, post‐acute COVID‐19 syndrome, SARS‐CoV‐2

## Abstract

Long COVID (LC) is a post‐acute infection syndrome affecting 5%–10% of individuals infected by SARS‐CoV‐2. Here, we aimed to study SARS‐CoV‐2 humoral immunity, immunological imprinting by endemic coronaviruses, and previous herpesvirus infections in LC. We included 47 LC patients and 41 controls who fully recovered from COVID‐19. We assessed IgG, IgA, and IgM antibody levels against SARS‐CoV‐2, seasonal coronaviruses, and herpesviruses using ELISAs and Microblot‐Array panels. Additionally, we performed PCR to detect viral RNA/DNA and evaluated anti‐nuclear autoantibodies linked to systemic autoimmune conditions. LC patients showed significantly reduced levels of SARS‐CoV‐2 anti‐spike IgG and IgA but increased levels of endemic coronaviruses OC43 and HKU1 anti‐spike IgG, suggesting immunological imprinting potentially driven by these coronaviruses. Furthermore, LC patients had higher levels of SARS‐CoV‐2 anti‐spike IgM compared to anti‐Spike IgG, possibly indicating impaired class switching. Interestingly, cytomegalovirus (CMV) p65 IgG levels were lower in LC patients and negatively correlated with fatigue severity. This study highlights immunological imprinting by seasonal coronaviruses and impaired antibody class switching as potential causes of SARS‐CoV‐2 immune escape and persistence in LC patients. Furthermore, our findings suggest an inverse association between CMV p65 IgG and fatigue severity in LC.

## 1 Introduction

While the coronavirus disease 2019 (COVID‐19) pandemic has officially ended, a substantial healthcare challenge persists as 5%–10% suffer from chronic symptoms after severe acute respiratory syndrome coronavirus 2 (SARS‐CoV‐2) infection. This post‐acute infectious disease condition, often referred to as “long COVID” (LC), is more often observed in females and can manifest through a wide range of symptoms [1, 2]. The most reported LC symptoms include fatigue, post‐exertional malaise (PEM), breathing difficulties, myalgia, headaches, and neurocognitive problems [3]. These symptoms can last from months to years, severely impacting overall health and quality of life [4, 5].

Several underlying pathophysiological mechanisms have been implicated in LC, including the persistence of SARS‐CoV‐2 viral particles or antigens, reactivation of herpesviruses, autoimmunity, and dysregulation of antiviral immune responses [6, 7, 8, 9, 10]. The persistence of SARS‐CoV‐2 could potentially be linked to ineffective neutralizing antibody responses, which may be the result of immunological imprinting (also known as the original antigenic sin) [11]. In immunological imprinting, B cells are re‐exposed to an antigen that is highly similar to an original antigen that B cells were first exposed to, where after the B cells mount a memory antibody response specific to the original antigen instead of the second antigen. Accordingly, these antibodies are less effective towards the second antigen, leading to viral escape [12]. With regard to SARS‐CoV‐2, B cells could be imprinted by the spike protein of seasonal coronaviruses OC43, HKU1, NL63, and 229E, which share high homology with the spike protein of SARS‐CoV‐2 [13, 14].

Another potential factor contributing to LC is the reactivation of herpesviruses. These viruses are characterized by their latent state inside the host after initial infection [15]. Under certain conditions, such as during immunosuppression, fever, tissue damage, and coinfection, these viruses can reactivate and cause disease [16]. Herpesvirus infections have been linked to an increased risk of systemic autoimmune diseases, often marked by anti‐nuclear antibodies (ANAs) [17, 18].

Here, we aimed to assess the role of immunological imprinting by endemic coronaviruses in LC. Furthermore, we sought to study whether antibody serostatus and reactivation of herpesviruses are related to LC and its specific clinical presentation. Finally, we investigated the potential involvement of autoimmunity in LC by examining a broad panel of clinically relevant ANA.

## 2 Methods

### 2.1 Study Population and Sample Collection

LC patients were recruited as participants of the “LARGO” randomized‐controlled trial conducted at Franciscus Hospital, as previously described [19]. The inclusion criteria were: between 18 and 70 years of age, PCR‐confirmed SARS‐CoV‐2 infection and at least two LC symptoms according to World Health Organization consensus criteria lasting < 1 year [20]. The exclusion criteria were prior admission to the intensive care unit due to COVID‐19, abnormal or changed chest radiography or pulmonary function test results, current acute SARS‐CoV‐2 infection or active systemic immunological disorders, current psychiatric disorders as diagnosed by a psychiatrist, use of oral/inhaled corticosteroids or other immune‐modulatory medication, current pregnancy or lactation, and allergy to milk or components of milk [19]. The present study included all LC patients from the LARGO trial for whom a baseline (i.e., before treatment intervention) serum sample was available. For comparison, a healthy control (HC) cohort of fully recovered COVID‐19 convalescent healthcare workers was used. These HC provided a heparinized plasma or serum sample during our previous COVID‐19 immunity studies. The inclusion criteria of this cohort were: between 18 and 65 years of age, PCR‐confirmed SARS‐CoV‐2 infection, and blood sample collected between November 2021 and July 2022 [21, 22].

For all participants, blood samples were obtained by venipuncture, whereafter the samples were stored at ‐80°C until analysis. Depending on sample availability per individual, serum or heparin plasma was used in the analysis. Additionally, LC patients reported their symptoms and completed the Cognitive Failures Questionnaire (CFQ) and Fatigue Assessment Score (FAS) assessments on the same day as blood collection.

The outcomes of this study included SARS‐CoV‐2 and endemic coronavirus‐specific antibody levels, SARS‐CoV‐2 RNA, herpesvirus DNA and antibody profiles, ANA profiles, and associations of these parameters with cognitive failure and fatigue.

The study followed the principles of the Declaration of Helsinki and all participants provided written informed consent.

### 2.2 Enzyme‐Linked Immunosorbent Assay (ELISA)

To assess Human Herpesvirus 6 (HHV‐6) antibodies, we utilized a commercial ELISA assay (KA1457, Abnova). Further, coronavirus‐specific antibodies were measured by in house designed ELISAs. For these, 50 µl per well of 0.5 µg/ml antigen solution in PBS was coated overnight at 4°C in 96‐well Maxisorp plates (442404, ThermoFisher). The following antigens were used: SARS‐CoV‐2 spike protein (40589‐V08H4, Sino Biological), spike subunit 1 (S1) (100730, BPS Bioscience), spike subunit 2 (S2) (S2N‐C52H5, ACRO Biosystems), and nucleocapsid protein (40588‐V08B, Sino Biological), as well as the spike protein of HCoV‐OC43 (40607‐V08B), HCoV‐HKU1 (40606‐V08B), HCoV‐NL63 (40604‐V08B), and HCoV‐229E (40605‐V08B), and spike S2 of HCoV‐OC43 (40607‐V08B1) and HCoV‐HKU1 (40021‐V08B, all Sino Biological). Plates were washed with 0.05% (vol/vol) Tween‐20 in PBS and blocked with 3% (mass/volume) low‐fat milk powder (T145, Carl Roth) in PBS for 1 h at room temperature (RT). Next, 1:50 diluted serum and plasma samples in 1% milk/PBS was incubated for 1 h at RT. After washing, 50 µl of 0.5–1.0 µg/ml HRP‐labeled monoclonal anti‐IgG (05‐4220, ThermoFisher), polyclonal anti‐IgA (411002, BioLegend), or polyclonal anti‐IgM (SA5‐10293, ThermoFisher) was added and incubated for 1 h at RT. After washing, 100 µl TMB substrate (34028, ThermoFisher) was incubated for 10 min in the dark at RT, followed by 100 µl/well of 1 M sulfuric acid. The optical density was measured at 450 and 620 nm for background signal correction using the ETI‐MAX 3000 (Diasorin). OD 450–620 values were transformed in natural log (Ln) to normalize the data by reducing skewness.

### 2.3 Virus Polymerase Chain Reaction (PCR)

Total nucleic acids from serum or plasma samples were extracted using the Emag system (BioMerieux) using the Emag blood protocol. PCR mixes were prepared by using 5 µl FastVirus mastermix (Applied Biosystems, ThermoFisher), 2 µl primer and probe mix for the various targets (EBV EBNA‐LP and BNRF1; CMV DNA polymerase and UL75; HHV‐6 DNA polymerase; HSV1 gG; HSV2 gD; VZV gene 38 and gB; SARS‐CoV‐2 E and N), 2 µl PCR graded water and 9 µl eluate or control were used. PCR cycles consisted of 5 min at 50°C, followed by 50 cycles of 15 s at 95°C and 60 s at 60°C performed at an ABI 7500 PCR cycler (ThermoFisher).

### 2.4 Microblot‐Array

Herpesvirus antibodies and anti‐nuclear antibodies (ANA) were assessed using the Microblot‐Array (MBA) kits for EBV IgG (EBGMA96), EBV IgM (EBMMA96), EBV IgA (EBAMA96), CMV IgG (CMGMA48), CMV IgM (CMMMA48), HSV1 + 2 IgG (HSGMA48), HSV1 + 2 IgM (HSMMA48), and ANA plus (ANApMA96, all from TestLine Clinical Diagnostics). Each well contained a nitrocellulose membrane on which multiple antigen spots were printed in triplicate. After pre‐wetting the membranes, 100 µl of 1:50 diluted serum or plasma was incubated on the membranes for 30 min, followed by 30 min incubation with 100 µl of alkaline phosphatase‐conjugated detection antibody. Finally, 100 µl of BCIP‐NBT substrate solution was incubated for 15 min, after which the membranes were washed with demineralized water. Membranes were dried for 30 min in an MBA dryer and were automatically imaged and analyzed using the MBA reader and corresponding software (ARCXIX096, TestLine Clinical Diagnostics). The images were manually inspected, where after antigen spots showing high background signal were excluded. Results were presented as units/ml (U/ml) and classified as negative ( < 185 U/ml) or positive ( ≥ 185 U/ml).

### 2.5 Statistical Analyses

All data are presented as medians with interquartile ranges (IQR). Statistical analyses were conducted using GraphPad Prism v10. Categorical data was compared using the Chi square test or Fisher's exact test if a group consisted of five or less values. Mann‐Whitney U tests were performed to compare two independent groups and Kruskal‐Wallis tests with Dunn's post hoc analysis were applied to compare between three independent groups. Spearman's rank correlations were used to assess associations. The *p*‐values were adjusted using the Benjamini‐Hochberg correction for multiple comparisons. Statistical tests were performed at a two‐tailed α‐level of 0.05.

## 3 Results

### 3.1 Study Population

A total of 47 LC patients and 41 HC with a previous SARS‐CoV‐2 infection were included in this study. The LC group comprised 18 (38.3%) males with a median age of 46 years (IQR 38–52), and the HC group included 9 (22.0%) males with a median age of 53 years (IQR 42–58) (Table 1). These demographics were not statistically significant between the two groups (*p* = 0.0972 for sex; *p* = 0.1154 for age). All participants had a confirmed SARS‐CoV‐2 infection through RT‐PCR or lateral flow antigen testing. Among the LC patients, 78.7% experienced a single SARS‐CoV‐2 infection, while 17.0% had two infections, and 2.1% had three infections. Similarly, 87.8% of the HC group had a single infection, 9.8% had two infections, and 2.4% had three infections, with no significant difference observed between the two study groups (*p* = 0.6785). Five LC patients required hospitalization during acute COVID‐19, whereas none of the HC were hospitalized (*p* = 0.0583). Except for three LC patients, all participants received at least one COVID‐19 vaccination. The distribution of the number of vaccinations per individual was comparable between the groups (*p* = 0.2572). The median time between the last SARS‐CoV‐2 infection and blood collection was significantly shorter in LC patients (280 days [IQR 142–331] than in HC (596 days [IQR 558–722], *p* < 0.0001).

**Table 1:** Characteristics of LC and HC cohorts. Data are presented as *n* (%) or median with IQR. NA, not available.

	Long COVID (*n* = 47)	Healthy controls (*n* = 41)	*p* value
Males	18 (38.3%)	9 (22.0%)	0.0972
Age	46 (38–52)	53 (42–58)	0.1154
Number of SARS‐CoV‐2 infections			0.6785
1	37 (78.7%)	36 (87.8%)	
2	8 (17.0%)	4 (9.8%)	
3	1 (2.1%)	1 (2.4%)	
Unknown	1 (2.1%)	0	
Hospital visit due to acute COVID‐19	5 (10.6%)	0	0.0583
Number of COVID‐19 vaccinations			0.2572
0	3 (6.4%)	0	
1	3 (6.4%)	4 (9.8%)	
2	17 (36.2%)	17 (41.5%)	
3	24 (51.1%)	13 (31.7%)	
Unknown	0	7 (17.1%)	
Median days in between last SARS‐CoV‐2 infection and blood collection	280 (142–331)	596 (558–722)	< 0.0001
Time window of blood collection	February 1, 2022–March 28, 2022	November 23, 2021–July 1, 2022	
FAS score		NA	
No fatigue (0–21)	0		
Mild to moderate fatigue (22–34)	28 (59.6%)		
Severe fatigue (35–50)	19 (40.4%)		
CFQ score		NA	
No cognitive failure (0–43)	17 (36.2%)		
Mild cognitive failure (44–54)	9 (19.1%)		
Severe cognitive failure (55–100)	21 (44.7%)		
Self‐reported symptoms		NA	
Fatigue	43 (91.5%)		
Cognitive disturbances	43 (91.5%)		
Muscle weakness/pain	17 (36.2%)		
Dyspnea	29 (61.7%)		
Headache	14 (29.8%)		
Chest pain	10 (21.3%)		
Sore throat	3 (6.38%)		
Depressive or anxiety symptoms	1 (2.1%)		

LC symptoms were evaluated using the FAS and the CFQ. Based on these questionnaires, all LC patients experienced fatigue, with 40.4% reporting severe fatigue, and 63.8% of LC patients experienced cognitive impairment, of whom 44.7% reported severe cognitive failure. LC patients also self‐reported various symptoms at the time of inclusion, of which fatigue (91.5%), cognitive disturbance (91.5%), and muscle pain/weakness (61.7%) were reported as the main symptoms.

### 3.2 LC Patients Have Decreased SARS‐CoV‐2 Spike‐Specific IgG and IgA But an Increased Proportion of Spike‐Specific IgM Relative to IgG

**Figure 1:**
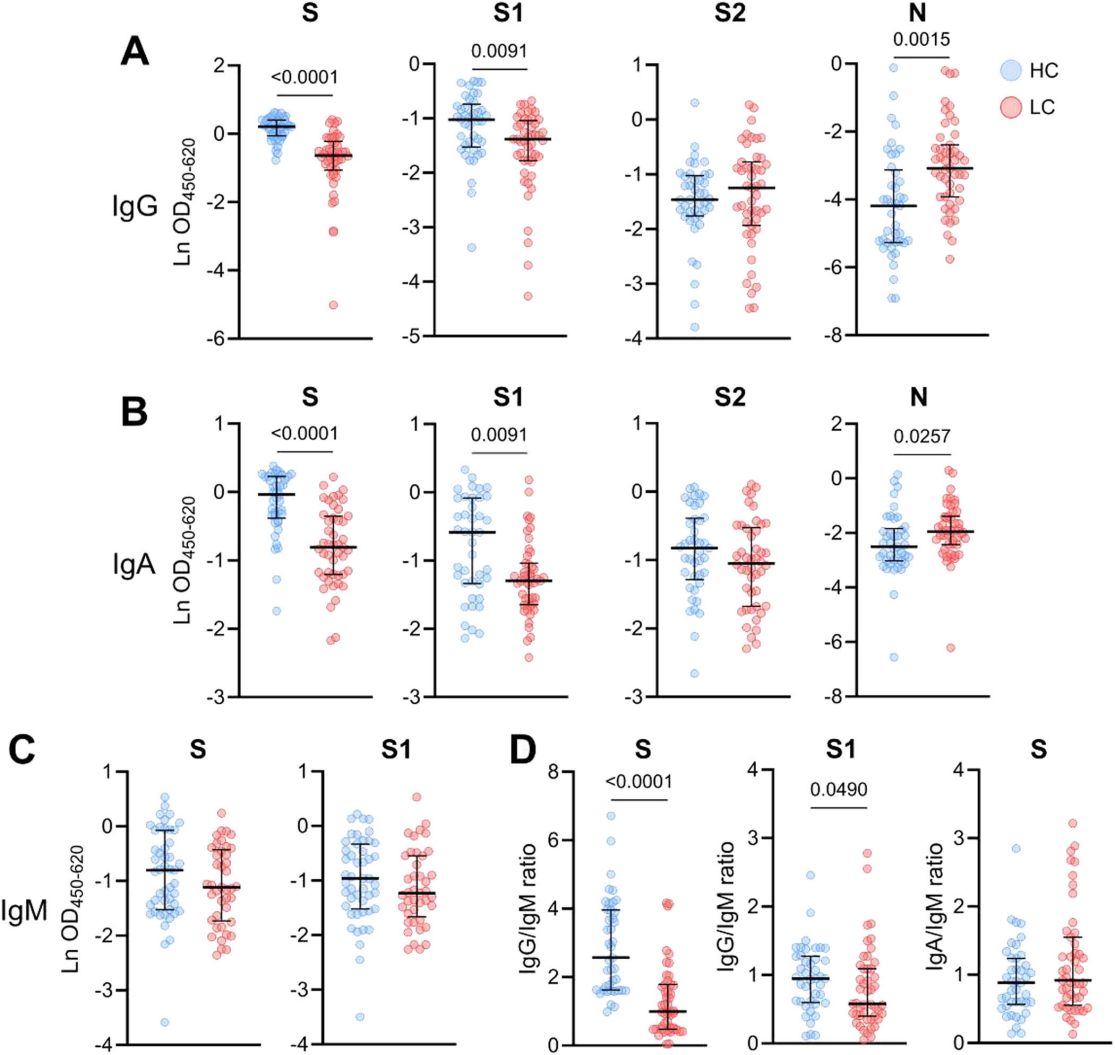
SARS‐CoV‐2‐specific IgG, IgA, and IgM antibody levels in HC and LC patients. (A) IgG and (B) IgA antibody levels against the full spike (S) protein, spike subunit 1 (S1), spike subunit 2 (S2), and nucleocapsid (N) protein of SARS‐CoV‐2. (C) IgM antibody levels against SARS‐CoV‐2 S and S1. (D) SARS‐CoV‐2 S and S1 IgG/IgM and S IgA/IgM ratios in HC and LC patients. OD450‐620 values were used for ratio calculations. Data are presented as median with IQR and statistical comparisons were made using the Mann‐Whitney U test, with *p*‐values adjusted for multiple comparisons using the Benjamini‐Hochberg method.

To evaluate the status of humoral immunity against SARS‐CoV‐2 in LC patients, we first measured the levels of IgG and IgA antibodies specific to SARS‐CoV‐2 in both LC patients and HC. LC patients exhibited significantly reduced levels of SARS‐CoV‐2 spike‐specific IgG and IgA antibodies compared to HC (both *p* < 0.0001) (Figure 1A,B). Further examination of the antibody response indicated that this reduction was primarily driven by antibodies targeting the S1 subunit of the SARS‐CoV‐2 spike protein. Specifically, S1‐specific IgG and IgA antibodies were markedly lower in LC patients (both *p* = 0.0091), while S2‐specific antibodies were comparable between the two groups. In contrast to the decreased spike‐specific antibody levels, nucleocapsid (N)‐specific IgG and IgA antibody levels were significantly elevated in LC patients, with a more pronounced difference for IgG antibodies (*p* = 0.0015 for IgG; *p* = 0.0257 for IgA). This increase in N‐specific antibodies may reflect a differential immune response in LC patients, potentially indicative of an elevated or prolonged exposure to viral antigens. Therefore, we investigated the presence of persistent SARS‐CoV‐2 RNA in LC patients by performing a SARS‐CoV‐2 RT‐PCR on LC serum. However, viral RNA was not detected in any of the LC patients (data not shown).

SARS‐CoV‐2 spike and S1‐specific IgM antibodies were comparable between LC patients and HC (Figure 1C). However, the spike‐specific IgG/IgM ratio was significantly lower in LC patients, indicating a relatively higher proportion of anti‐spike IgM compared to anti‐spike IgG in this group (*p* < 0.0001). Although less pronounced, a similar trend was observed for the S1‐specific IgG/IgM ratio, which was also significantly lower in LC patients (*p* = 0.0490). In contrast, the spike‐specific IgA/IgM ratios did not differ between HC and LC patients, suggesting comparable distributions of these antibody isotypes across the two groups.

To determine whether these positive findings were related to LC symptomatology, we assessed correlations between these findings and FAS and CFQ scores. SARS‐CoV‐2 antibodies were not associated with fatigue or cognitive failure severity (Supporting Information Table 1). Additionally, we determined whether these antibody responses were different in LC patients infected with the Alpha, Delta, and Omicron variant (Supporting Information Figure 1). Omicron‐infected LC patients had higher anti‐spike IgG than Alpha‐infected LC patients, likely caused by the shorter time window between infection and blood collection in the Omicron group. No other significant differences in antibody responses were observed between the variant groups.

### 3.3 LC Patients Have Elevated HKU1 and OC43 Anti‐Spike IgG Antibody Levels

**Figure 2:**
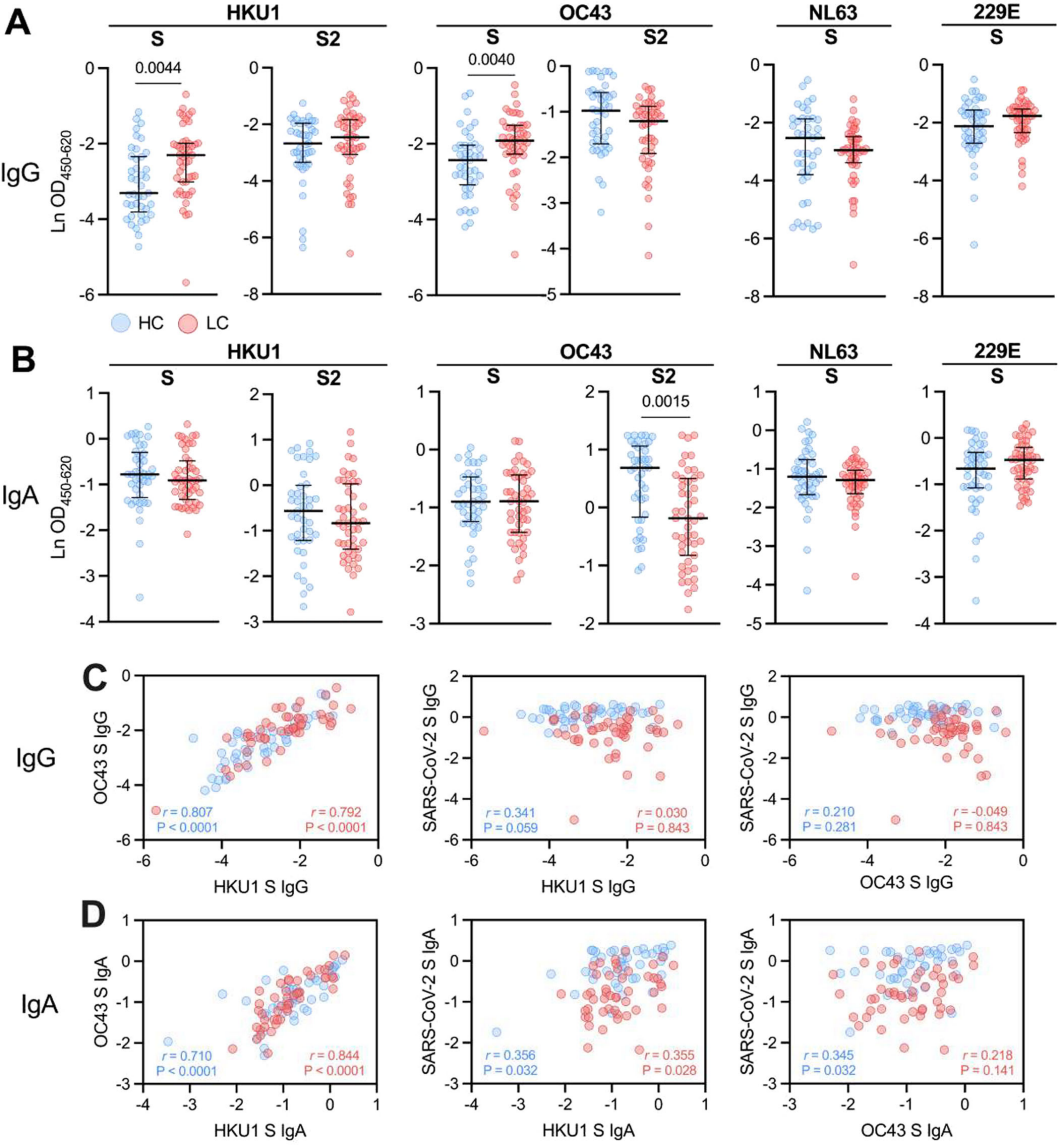
Seasonal coronavirus antibody levels in HC and LC patients. (A) IgG and (B) IgA antibody titers against full spike (S) protein and spike subunit 2 (S2) of endemic betacoronaviruses HKU1 and OC43, as well as the alphacoronaviruses NL63 and 229E. Data are presented as median with IQR and comparisons between groups were performed with the Mann‐Whitney U test. (C and D) Associations between OC43, HKU1, and SARS‐CoV‐2 spike‐specific antibody titers in LC and HC groups, assessed by Spearman's rank correlation testing for (C) IgG and (D) IgA. All *p* values were adjusted for multiple comparisons using the Benjamini‐Hochberg method.

To explore the potential role of immunological imprinting by seasonal coronaviruses in the development of LC, we analyzed IgG and IgA antibody levels against the spike proteins of seasonal coronaviruses HKU1, OC43, NL63, and 229E in both groups. The analysis revealed that LC patients exhibited significantly elevated IgG antibody levels against the spike proteins of both HKU1 and OC43 compared to HC (*p* = 0.0044 for HKU1; *p* = 0.0040 for OC43), without differences in S2‐specific IgG levels between the groups (Figure 2A). In contrast to IgG, HKU1 and OC43 S‐specific IgA levels were comparable between LC patients and HC (Figure 2B), and OC43 S2‐specific IgA levels were increased in LC patients (*p* = 0.0015). Furthermore, NL63 and 229E spike‐specific antibody levels for both IgG and IgA isotypes were similar between the two groups. HKU1 or OC43 anti‐spike antibody levels were not associated with fatigue or cognitive failure severity (Supporting Information Table 1). No differences in HKU1‐ or OC43‐specific anti‐spike IgG levels were observed between LC patients infected with the Alpha, Delta, or Omicron variant (Supporting Information Figure 2).

To characterize cross‐reactivity between anti‐spike antibodies targeting the different betacoronaviruses, we assessed the associations between the anti‐spike antibody levels. A strong positive correlation was observed between IgG levels against the spike proteins of HKU1 and OC43 in both groups, but no significant correlations were found between the IgG levels to these betacoronaviruses and SARS‐CoV‐2 spike‐specific IgG (Figure 2C). For IgA titers, a similar strong positive correlation was observed between the spike‐specific IgA titers of HKU1 and OC43 (Figure 2D). Also, there was a weak significant correlation between HKU1 or OC43 and SARS‐CoV‐2 spike‐specific IgA levels.

### 3.4 LC Patients Have Decreased CMV Antibody Levels Which Associates With Fatigue Severity

To investigate the potential role of herpesvirus reactivation in the development of LC, we first assessed the presence of DNA of EBV, CMV, HSV‐1, HSV‐2, VZV, and HHV‐6 in the serum of LC patients. DNA of these herpesviruses was undetectable in all samples, except for HHV‐6 DNA, which was identified in one LC patient (2.1%; data not shown).

**Figure 3:**
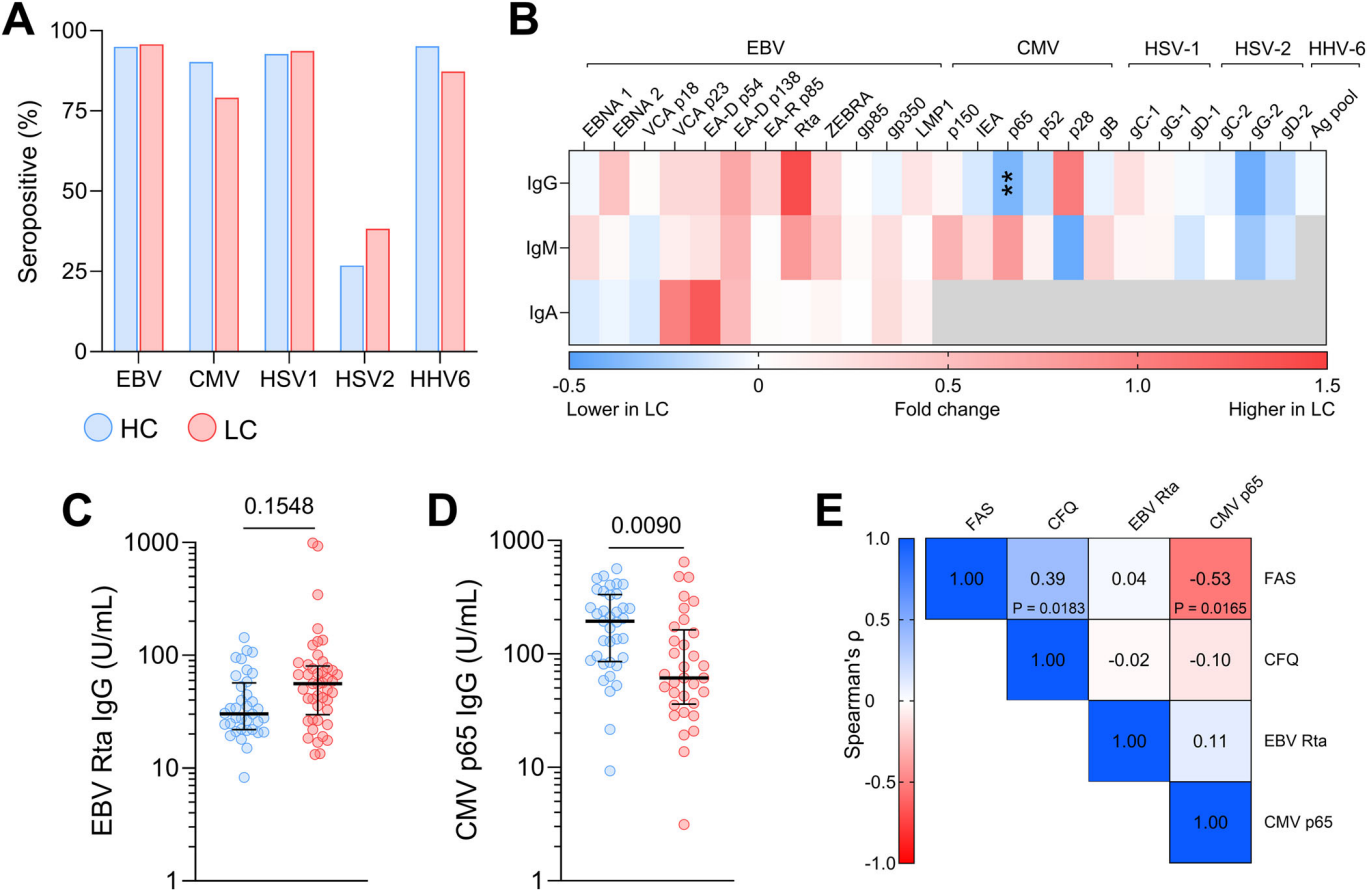
Herpesvirus antibody levels in HC and LC patients. (A) Percentage of seropositive HC and LC patients, determined by the presence of detectable IgG antibodies against at least one antigen from each herpesvirus. (B) Heatmap showing the average percentage differences in IgG, IgM, and IgA antibody titers against herpesviruses between seropositive HC and LC patients. Grey fields in the heatmap were not assessed. (C) Comparison of EBV Rta and (D) CMV p65 IgG titers between HC and LC patients, analyzed using the Mann‐Whitney U test. (E) Spearman's rank correlations between the Fatigue Assessment Scale (FAS), Cognitive Failure Questionnaire (CFQ), EBV Rta IgG titers, and CMV p65 IgG titers in LC patients. All *p*‐values were adjusted using the Benjamini‐Hochberg method for multiple comparisons.

Subsequently, we evaluated antibody responses against EBV, CMV, HSV‐1, HSV‐2, and HHV‐6. Seroprevalence rates for all viruses were comparable between HC and LC patients (Figure 3A). For seropositive individuals, we conducted a quantitative assessment of IgG, IgM, and IgA antibodies specific to various herpesvirus antigens. EBV‐specific antibodies generally tended to be higher in LC patients, although none reached statistical significance (Figure 3B). For example, IgG antibodies binding the EBV reactivation‐associated transcription factor Rta were elevated by 139% in LC patients compared to HC (Figure 3C). In contrast, CMV‐specific IgG antibodies predominantly showed a trend towards lower levels in LC patients, with significantly reduced IgG against the p65 antigen (*p* = 0.0090) (Figure 3D). No clear patterns were observed in the antibody levels against HSV‐1, HSV‐2, and HHV‐6 between the HC and LC groups. To further explore the clinical relevance of these serological findings, we assessed the correlation between antibody levels against EBV Rta and CMV p65, and clinical features of LC, including fatigue and cognitive impairment as measured by the FAS and CFQ, respectively. A significant negative correlation was found between CMV p65‐specific IgG levels and FAS scores (r = − 0.53, *p* = 0.0165), indicating that lower CMV IgG antibody levels were associated with increased fatigue severity in LC patients (Figure 3E).

### 3.5 LC Patients Do Not Have a Distinct Anti‐Nuclear Autoantibody Profile

**Figure 4:**
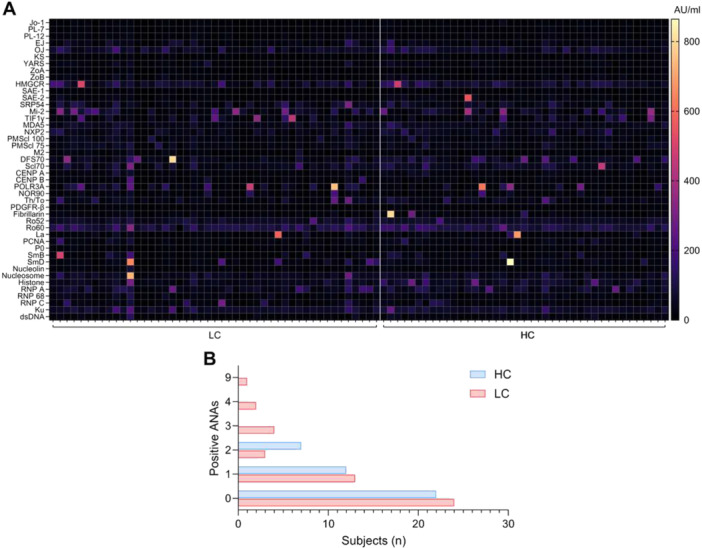
ANA profiles in HC and LC patients. (A) Heatmap displaying the concentrations of various IgG ANAs. Each row represents a different autoantibody type, and each column corresponds to an individual LC patient or HC. (B) The total number of detectable autoantibodies per individual.

To investigate whether LC is associated with increased levels of antinuclear antibodies (ANAs), potentially contributing to increased understanding on disease mechanisms or serving as biomarkers, we analyzed a panel of 44 ANAs which are highly associated with the most common systemic autoimmune diseases. At least one ANA was detected in 46.3% of HC and 48.9% of LC patients but we were unable to identify specific ANA or distinct ANA profiles unique to LC patients (Figure 4A). Although the number of detectable ANAs per individual was not statistically different between the groups, seven LC patients versus none of the HC tested positive for 3 or more positive ANAs (Figure 4B).

## 4 Discussion

The mechanisms driving LC are complex and multifaceted, and they likely involve a combination of factors such as viral persistence, immune dysregulation, autoimmunity, and reactivation of latent viruses [6, 7]. In this study, we investigated whether these mechanisms could be confirmed in LC patients.

Immunological imprinting, or original antigenic sin, was first described in the 1950s in the context of influenza vaccination, but has since been linked to various other pathogens, including SARS‐CoV‐2 [23]. Imprinting by seasonal coronaviruses has been suggested to contribute to impaired clearance of SARS‐CoV‐2 and potentially influence the severity of acute COVID‐19 [24]. These seasonal coronaviruses, which typically cause mild upper respiratory tract infections, are categorized into alphacoronaviruses (NL63 and 229) and betacoronaviruses (OC43 and HKU1). As a betacoronavirus, SARS‐CoV‐2 shares high spike protein homology with OC43 and HKU1 (40% C‐terminal region and 25% N‐terminal region sequence identity) [13, 14]. This considerable homology could lead to imprinting of B cells, accounting for increased antibody levels against OC43 and HKU1 and reduced SARS‐CoV‐2‐specific IgG levels in LC patients. In line with these results, two previous studies that included 18 and 17 LC patients also reported reduced SARS‐CoV‐2 spike antibody levels and diminished Fcγ receptor binding of these antibodies, alongside elevated endemic coronaviruses spike antibody levels [25, 26]. Thus, immunological imprinting may predominantly drive the production of antibodies that target conserved regions shared with endemic coronaviruses rather than unique epitopes of SARS‐CoV‐2, resulting in suboptimal neutralization.

Immunological imprinting may also interfere with the generation of high‐affinity antibody responses through impaired isotype switching. Normally, 2 weeks after first antigen exposure, IgM‐producing B cells undergo class‐switch recombination, enabling production of higher‐affinity IgG with distinct effector functions [27]. After class‐switching, IgM production ceases, leaving low to undetectable SARS‐CoV‐2‐specific IgM levels in the months following SARS‐CoV‐2 infection [28, 29]. The combination of increased SARS‐CoV‐2 anti‐spike IgM and decreased anti‐spike IgG in our LC cohort may reflect impaired class switching, potentially as a cause of immunological imprinting. For instance, pre‐existing memory B cells specific to endemic coronaviruses might compete with naive B cells for T cell help, as memory B cells typically express higher levels of costimulatory molecules than naive B cells. Accordingly, reduced T cell engagement with naive B cells could limit proliferation and differentiation into SARS‐CoV‐2‐specific plasma cells or memory B cells [30, 31]. As impaired isotype switching may hinder the transition from low‐affinity IgM to high‐affinity IgG responses, this may further reduce the effectiveness of the antibody response. Notably, impaired class switching has also been observed in systemic lupus erythematosus (SLE) patients after COVID‐19 vaccination, suggesting similarities in immune dysregulation between SLE and LC [32].

The inadequate SARS‐CoV‐2 antibody response likely contributes to impaired viral clearance in LC patients, allowing the virus or viral antigens to persist and drive chronic inflammation [11]. Several studies have detected SARS‐CoV‐2 RNA and proteins in blood, stool, and tissue biopsy samples from LC patients for weeks to months after the acute phase of infection [33, 34, 35, 36, 37, 38, 39, 40]. Moreover, one study that applied a highly sensitive digital droplet PCR specifically linked SARS‐CoV‐2 persistence with LC symptomatology [41]. However, replication‐competent virus has not been detected in LC patients, as studies to date have only identified viral RNA or proteins. In our study, SARS‐CoV‐2 RNA was not detected in serum, possibly because the virus persists primarily in tissues with minimal shedding into the circulation, requiring more sensitive detection techniques. Nonetheless, the elevated nucleocapsid IgG levels in LC patients suggests persistence of this protein and possibly of viral particles, although this may also reflect the shorter interval between infection and sampling in the LC cohort.

Immunological imprinting has also been observed across SARS‐CoV‐2 variants, as shown by the dominance of Wuhan strain‐specific memory B cells following Omicron XBB1.5 booster vaccination [42]. Thus, immunological imprinting might also have occurred following exposure to the spike protein of an earlier SARS‐CoV‐2 variant, either through infection or vaccination, and subsequently being infected with a new variant, leading to immune escape and persistence of this variant. This could explain the occurrence of new‐onset LC cases following SARS‐CoV‐2 reinfections [43, 44]. Unfortunately, we were unable to test imprinting by previous variants due to variant heterogeneity among participants in the LC and HC groups. Nevertheless, we attempted to determine whether HKU1 and OC43 induced imprinting differed between LC patients infected with distinct SARS‐CoV‐2 variants but were unable to associate these responses with any particular variant.

Given the potential link between herpesvirus reactivation and LC pathophysiology, we also explored this aspect in our cohort. Reactivation of herpesviruses has been widely documented during the acute phase of COVID‐19, with more severe cases showing higher reactivation rates [45]. Possibly, this reactivation might be extended into the post‐acute phase, as recent studies reported ongoing herpesvirus reactivation in LC patients. In particular an active EBV [46, 47] and HHV‐6 [48, 49] infection has already been related to myalgic encephalomyelitis/chronic fatigue syndrome (ME/CFS), a condition with highly similar clinical features as LC [50]. Reactivation of EBV and HHV6 may contribute to LC through several mechanisms, such as by molecular mimicry where antibodies against EBV proteins cross‐react with human proteins [51, 52, 53, 54, 55].

In LC cohort studies, increased EBV antibody levels were observed in 66.7% of cases, as indicated by EA‐D IgG or VCA IgM positivity, indicative for reactivation [56]. Other studies detected DNA of EBV, HHV‐6, or both in saliva and mucous membranes in 72.3% of LC patients and EBV DNA in throat washings of 50% of LC patients, whereas EBV viremia was observed in none, 2.3%, and 14% of LC patients [35, 57, 58, 59]. In our study, all LC patients tested negative for EBV, CMV, VZV, HSV‐1, HSV‐2, and HHV‐6 DNA, except for one individual who tested positive for HHV‐6. This suggests that viremia was no longer detectable during blood collection (i.e., mostly beyond 9 months after SARS‐CoV‐2 infection). Nevertheless, it remains plausible that reactivation occurred only during or shortly after the acute SARS‐CoV‐2 infection, as other studies have indicated [45]. Therefore, we also analyzed herpesvirus‐specific antibody profiles in LC patients and compared them with those in HC. Although herpesvirus DNA detection remains the gold standard for assessing reactivation, increased antibody levels may also indicate a recent infection or reactivation event, despite being a less sensitive and specific measure. Interestingly, while EBV IgG levels were not statistically higher in LC patients, we observed a trend toward increased average IgG levels against most EBV antigens, including EA‐D and Rta, which are typically associated with recent active infection [60, 61]. However, unlike some previous studies [56, 59], our findings did not reveal an association between EBV antibody levels and fatigue or cognitive disturbances.

Surprisingly, CMV p65 IgG levels were decreased and negatively correlated with fatigue severity in LC patients. A recent study involving 208 LC patients reported similar findings as individuals with a prior CMV infection were less likely to specifically develop neurocognitive symptoms. It remains uncertain how CMV or CMV‐specific antibodies could be related to LC symptomatology, but it is speculated that a stronger adaptive immune response against SARS‐CoV‐2 is mounted in individuals with a more recent CMV infection since this was also observed in influenza vaccinees [59]. Nevertheless, further studies should validate above findings in independent LC cohorts.

To investigate autoimmunity in our LC cohort, we employed an elaborate ANA panel containing nuclear antigens associated with systemic autoimmune diseases. ANAs can arise following heightened type I interferon (IFN) production in response to viral infections, which may explain why we detected ANAs in several healthy controls [62]. This aligns with previous studies reporting increased ANA levels in COVID‐19 patients and convalescents, regardless of LC development [63, 64]. Persistent SARS‐CoV‐2 infection may sustain elevated type I IFN levels and ongoing ANA production. However, unlike a few previous studies that reported increased ANAs in LC patients up to 12 months post SARS‐CoV‐2 infection, we could not confirm such elevations in our cohort [64, 65].

A strength of this study is the confirmation of previously reported immunological imprinting by seasonal coronaviruses in a larger, independent cohort of LC patients with comparison to previously SARS‐CoV‐2 infected patients without LC. Additionally, while most studies have either focused on the detection of DNA or the presence of antibodies for a limited number of herpesviruses, we employed both approaches to comprehensively assess most human herpesviruses. However, several limitations should be acknowledged. HC samples were collected a median of 316 days later post‐SARS‐CoV‐2 infection than LC patient samples (596 days vs. 280 days, respectively), possibly affecting results as different SARS‐CoV‐2 variants circulated during this interval. Also, we were restricted in the extent of antibody analyses. Detailed analysis of B cell clones, for example through single‐cell sequencing and antibody binding assays, is essential to understand how immunological imprinting and impaired isotype switching shape the antibody responses in LC.

In conclusion, this study underscores the potential involvement of immunological imprinting by seasonal coronaviruses and impaired class switching of SARS‐CoV‐2 antibodies in LC. These mechanisms could lead to decreased SARS‐CoV‐2 anti‐spike IgG levels, resulting in impaired SARS‐CoV‐2 clearance. Our comprehensive analysis of multiple herpesviruses further suggests a potential involvement of CMV or CMV p65‐specific IgG antibodies with LC symptoms. These findings provide valuable insights into potential mechanisms of LC, warranting further investigation with in‐depth analyses.

## Author Contributions

W. Ashwin Mak and David S. Y. Ong conceptualized the study. W. Ashwin Mak, Daphne Wapperom, Anne‐Lotte Redel, and Pieter W. Smit were involved in investigation and data curation. W. Ashwin Mak, Hung‐Jen Chen, Jeroen den Dunnen, and David S. Y. Ong conducted formal analyzes. W. Ashwin Mak visualized the data. Anne‐Lotte Redel, Johannes G. M. Koeleman, Wai‐Kwan Lam‐Tse, Tom van der Poll, Hung‐Jen Chen, Jeroen den Dunnen, Gert‐Jan Braunstahl, and David S. Y. Ong provided feedback and guidance supporting and interpreting analysis. David S. Y. Ong and Gert‐Jan Braunstahl provided supervision. W. Ashwin Mak wrote the original draft and all authors contributed, edited, and approved the final article.

## Conflicts of Interest

The authors declare no conflicts of interest.

## Supporting information


**Supplementary Table 1:** Associatons between anti‐coronavirus antibodies and fatigue and cognitive failure severity in LC patients. **Supplementary Figure 1:** SARS‐CoV‐2‐specific IgG, IgA, and IgM antibody levels in LC patients infected with the Alpha, Delta, or Omicron variant. **Supplementary Figure 2:** HKU1 and OC43 spike‐specific antibody levels in LC patients infected with the Alpha, Delta, or Omicron variant.

## Data Availability

The data that support the findings of this study are available from the corresponding author upon reasonable request.

## References

[1] A. V. Ballering , S. K. R. Van Zon , T. C. Olde Hartman , and J. G. M. Rosmalen , “Persistence of Somatic Symptoms After COVID‐19 in the Netherlands: An Observational Cohort Study,” Lancet 400, no. 10350 (2022): 452–461, 10.1016/s0140-6736(22)01214-4. 35934007 PMC9352274

[2] Z. Fang , R. Ahrnsbrak , and A. Rekito , “Evidence Mounts That About 7% of US Adults Have Had Long COVID,” Journal of the American Medical Association (Chicago, IL) 332 (2024): 5, 10.1001/jama.2024.11370. 38848092

[3] L. L. O'Mahoney , A. Routen , C. Gillies , et al., “The Prevalence and Long‐Term Health Effects of Long Covid Among Hospitalised and Non‐Hospitalised Populations: A Systematic Review and Meta‐Analysis,” EClinicalMedicine 55 (2023): 101762, 10.1016/j.eclinm.2022.101762. 36474804 PMC9714474

[4] Y. Kim , S. Bae , H. H. Chang , and S. W. Kim , “Long COVID Prevalence and Impact on Quality of Life 2 Years After Acute Covid‐19,” Scientific Reports 13, no. 1 (2023): 11207, 10.1038/s41598-023-36995-4. 37433819 PMC10336045

[5] E. W. Ely , L. M. Brown , and H. V. Fineberg , “Long Covid Defined,” New England Journal of Medicine 391, no. 18 (2024): 1746–1753, 10.1056/NEJMsb2408466. 39083764 PMC11687645

[6] H. E. Davis , L. McCorkell , J. M. Vogel , and E. J. Topol , “Long COVID: Major Findings, Mechanisms and Recommendations,” Nature Reviews Microbiology 21, no. 3 (2023): 133–146, 10.1038/s41579-022-00846-2. 36639608 PMC9839201

[7] D. M. Altmann , E. M. Whettlock , S. Liu , D. J. Arachchillage , and R. J. Boyton , “The Immunology of Long COVID,” Nature Reviews Immunology 23, no. 10 (2023): 618–634, 10.1038/s41577-023-00904-7. 37433988

[8] B. Krishna , M. Wills , and N. Sithole , “Long COVID: What is Known and What Gaps Need to be Addressed,” British Medical Bulletin 147, no. 1 (2023): 6–19, 10.1093/bmb/ldad016. 37434326 PMC10502447

[9] Z. A. Sherif , C. R. Gomez , T. J. Connors , T. J. Henrich , and W. B. Reeves , “Pathogenic Mechanisms of Post‐Acute Sequelae of SARS‐CoV‐2 Infection (PASC),” eLife 12 (2023): e86002, 10.7554/eLife.86002. 36947108 PMC10032659

[10] M. J. Peluso and S. G. Deeks , “Mechanisms of Long Covid and the Path Toward Therapeutics,” Cell 187, no. 20 (2024): 5500–5529, From NLM Medline, 10.1016/j.cell.2024.07.054. 39326415 PMC11455603

[11] D. L. Kolson , “Can Immunological Imprinting Drive Neurological Dysfunction in Long Covid?,” Brain 146, no. 10 (2023): 3960–3962, 10.1093/brain/awad307. 37681523 PMC11004940

[12] A. Vatti , D. M. Monsalve , Y. Pacheco , C. Chang , J. M. Anaya , and M. E. Gershwin , “Original Antigenic Sin: A Comprehensive Review,” Journal of Autoimmunity 83 (2017): 12–21, 10.1016/j.jaut.2017.04.008. 28479213

[13] J. Braun , L. Loyal , M. Frentsch , et al., “SARS‐CoV‐2‐reactive T Cells In Healthy Donors and Patients With Covid‐19,” Nature 587, no. 7833 (2020): 270–274, From NLM Medline, 10.1038/s41586-020-2598-9. 32726801

[14] J. Hicks , C. Klumpp‐Thomas , H. Kalish , et al., “Serologic Cross‐Reactivity of SARS‐CoV‐2 With Endemic and Seasonal Betacoronaviruses,” Journal of Clinical Immunology 41, no. 5 (2021): 906–913, 10.1007/s10875-021-00997-6. 33725211 PMC7962425

[15] J. I. Cohen , “Herpesvirus Latency,” Journal of Clinical Investigation 130, no. 7 (2020): 3361–3369, 10.1172/JCI136225. 32364538 PMC7324166

[16] T. Stoeger and H. Adler , “Novel” Triggers of Herpesvirus Reactivation and Their Potential Health Relevance,” Frontiers in Microbiology 9 (2019): 3207, 10.3389/fmicb.2018.03207. 30666238 PMC6330347

[17] G. Houen and N. H. Trier , “Epstein‐Barr Virus and Systemic Autoimmune Diseases,” Frontiers in Immunology 11 (2021): 587380, 10.3389/fimmu.2020.587380. 33488588 PMC7817975

[18] W. H. Robinson , S. Younis , Z. Z. Love , L. Steinman , and T. V. Lanz , “Epstein‐Barr Virus as a Potentiator of Autoimmune Diseases,” Nature Reviews Rheumatology 20, no. 11 (2024): 729–740, 10.1038/s41584-024-01167-9. 39390260

[19] A. L. Redel , F. Miry , M. E. Hellemons , L. M. A. Oswald , and G. J. Braunstahl , “Effect of Lactoferrin Treatment on Symptoms and Physical Performance in Long Covid Patients: A Randomised, Double‐Blind, Placebo‐Controlled Trial,” ERJ Open Research 10, no. 4 (2024): 00031‐2024, 10.1183/23120541.00031-2024. 39076533 PMC11284587

[20] J. A. Appiah , A. L. Cramond , V. De , et al., A Clinical Case Definition for Post COVID‐19 Condition in Children and Adolescents by Expert Consensus (World Health Organization, 2023).

[21] M. R. Faas , W. A. Mak , H. Y. Markus , et al., “Dynamics of Antibody and T Cell Immunity Against SARS‐CoV‐2 Variants of Concern and the Impact of Booster Vaccinations in Previously Infected and Infection‐Naïve Individuals,” Vaccines 10, no. 12 (2022): 2132, 10.3390/vaccines10122132. 36560542 PMC9784197

[22] W. A. Mak , W. Visser , M. Van Der Vliet , H. Y. Markus , J. G. M. Koeleman , and D. S. Y. Ong , “Ancestral SARS‐CoV‐2 and Omicron BA.5‐specific Neutralizing Antibody and T‐Cell Responses After Omicron Bivalent Booster Vaccination in Previously Infected and Infection‐Naive Individuals,” Journal of Medical Virology 95, no. 8 (2023): e28989, 10.1002/jmv.28989. 37565645

[23] A. S. Monto , R. E. Malosh , J. G. Petrie , and E. T. Martin , “The Doctrine of Original Antigenic Sin: Separating Good From Evil,” Journal of Infectious Diseases 215, no. 12 (2017): 1782–1788, 10.1093/infdis/jix173. 28398521 PMC5853211

[24] M. Aguilar‐Bretones , B. M. Westerhuis , M. P. Raadsen , et al., “Seasonal Coronavirus‐Specific B Cells With Limited SARS‐CoV‐2 Cross‐Reactivity Dominate the IgG Response in Severe Covid‐19,” Journal of Clinical Investigation 131, no. 21 (2021): eux150613, 10.1172/JCI150613. PMC8553556 34499051

[25] M. Spatola , N. Nziza , W. Jung , et al., “Neurologic Sequelae of COVID‐19 are Determined by Immunologic Imprinting From Previous Coronaviruses,” Brain 146, no. 10 (2023): 4292–4305, 10.1093/brain/awad155. 37161609 PMC11004923

[26] J. D. Herman , C. Atyeo , Y. Zur , et al., “Humoral Immunity to an Endemic Coronavirus is Associated With Postacute Sequelae of COVID‐19 in Individuals With Rheumatic Diseases,” Science Translational Medicine 15, no. 712 (2023): eadf6598, 10.1126/scitranslmed.adf6598. 37672567 PMC10764151

[27] K. Yu and M. R. Lieber , “Current Insights Into the Mechanism of Mammalian Immunoglobulin Class Switch Recombination,” Critical Reviews in Biochemistry and Molecular Biology 54, no. 4 (2019): 333–351, 10.1080/10409238.2019.1659227. 31509023 PMC6856442

[28] N. Ortega , M. Ribes , M. Vidal , et al., “Seven‐Month Kinetics of SARS‐CoV‐2 Antibodies and Role of Pre‐Existing Antibodies to Human Coronaviruses,” Nature Communications 12, no. 1 (2021): 4740, 10.1038/s41467-021-24979-9. PMC8346582 34362897

[29] R. Rubio , D. Macià , D. Barrios , et al., “High‐Resolution Kinetics and Cellular Determinants of SARS‐CoV‐2 Antibody Response over Two Years After COVID‐19 Vaccination,” Microbes and Infection 27 (2025): 105423, 10.1016/j.micinf.2024.105423. 39299570

[30] T. Inoue and T. Kurosaki , “Memory B Cells,” Nature Reviews Immunology 24, no. 1 (2024): 5–17, 10.1038/s41577-023-00897-3. 37400644

[31] S. G. Tangye , D. T. Avery , E. K. Deenick , and P. D. Hodgkin , “Intrinsic Differences in the Proliferation of Naive and Memory Human B Cells as a Mechanism for Enhanced Secondary Immune Responses,” Journal of Immunology 170, no. 2 (2003): 686–694, 10.4049/jimmunol.170.2.686. 12517929

[32] G. Montamat , C. E. Meehan , H. F. Bradford , et al., “Reduced Response to SARS‐CoV‐2 Vaccination is Associated With Impaired Immunoglobulin Class Switch Recombination in SLE Patients,” Clinical and Experimental Immunology 219 (2024): uxae119, 10.1093/cei/uxae119. PMC11773804 39658056

[33] V. Craddock , A. Mahajan , L. Spikes , et al., “Persistent Circulation of Soluble and Extracellular Vesicle‐Linked Spike Protein in Individuals With Postacute Sequelae of COVID‐19,” Journal of Medical Virology 95, no. 2 (2023): e28568, 10.1002/jmv.28568. 36756925 PMC10048846

[34] D. Goh , J. C. T. Lim , S. B. Fernaíndez , et al., “Case Report: Persistence of Residual Antigen and RNA of the SARS‐CoV‐2 Virus in Tissues of Two Patients With Long Covid,” Frontiers in Immunology 13 (2022): 939989, 10.3389/fimmu.2022.939989. 36131932 PMC9483160

[35] Y. Su , D. Yuan , D. G. Chen , et al., “Multiple Early Factors Anticipate Post‐Acute COVID‐19 Sequelae,” Cell 185, no. 5 (2022): 881–895.e20, 10.1016/j.cell.2022.01.014. 35216672 PMC8786632

[36] Z. Swank , Y. Senussi , Z. Manickas‐Hill , et al., “Persistent Circulating Severe Acute Respiratory Syndrome Coronavirus 2 Spike is Associated With Post‐Acute Coronavirus Disease 2019 Sequelae,” Clinical Infectious Diseases 76, no. 3 (2023): e487–e490, 10.1093/cid/ciac722. 36052466 PMC10169416

[37] F. Tejerina , P. Catalan , C. Rodriguez‐Grande , et al., “Post‐COVID‐19 Syndrome. SARS‐CoV‐2 RNA Detection in Plasma, Stool, and Urine In Patients With Persistent Symptoms After COVID‐19,” BMC Infectious Diseases 22, no. 1 (2022): 211, 10.1186/s12879-022-07153-4. 35240997 PMC8892394

[38] D. Viszlayová , M. Sojka , S. Dobrodenková , et al., “SARS‐CoV‐2 RNA in the Cerebrospinal Fluid of a Patient With Long Covid,” Therapeutic Advances in Infectious Disease 8 (2021): 20499361211048572, 10.1177/20499361211048572. 34659752 PMC8511908

[39] C. Schultheiß , E. Willscher , L. Paschold , et al., “Liquid Biomarkers of Macrophage Dysregulation and Circulating Spike Protein Illustrate the Biological Heterogeneity in Patients With Post‐Acute Sequelae of COVID‐19,” Journal of Medical Virology 95, no. 1 (2023): e28364, 10.1002/jmv.28364. 36458566 PMC9878213

[40] A. Zollner , R. Koch , A. Jukic , et al., “Postacute COVID‐19 is Characterized by Gut Viral Antigen Persistence in Inflammatory Bowel Diseases,” Gastroenterology 163, no. 2 (2022): 495–506.e8, 10.1053/j.gastro.2022.04.037. 35508284 PMC9057012

[41] W. Zuo , D. He , C. Liang , et al., “The Persistence of SARS‐CoV‐2 in Tissues and its Association With Long Covid Symptoms: A Cross‐Sectional Cohort Study in China,” Lancet Infectious Diseases 24, no. 8 (2024): 845–855, 10.1016/S1473-3099(24)00171-3. 38663423

[42] M. A. Tortorici , A. Addetia , A. J. Seo , et al., “Persistent Immune Imprinting Occurs After Vaccination With the COVID‐19 XBB.1.5 mRNA Booster in Humans,” Immunity 57, no. 4 (2024): 904–911.e4, 10.1016/j.immuni.2024.02.016. 38490197 PMC12360627

[43] O. Y. Bello‐Chavolla , C. A. Fermín‐Martínez , D. Ramírez‐García , et al., “Prevalence and Determinants of Post‐Acute Sequelae After SARS‐CoV‐2 Infection (Long COVID) Among Adults in Mexico During 2022: A Retrospective Analysis of Nationally Representative Data,” Lancet Regional Health ‐ Americas 30 (2024): 100688, 10.1016/j.lana.2024.100688. 38327277 PMC10847769

[44] B. Bowe , Y. Xie , and Z. Al‐Aly , “Acute and Postacute Sequelae Associated With SARS‐CoV‐2 Reinfection,” Nature Medicine 28, no. 11 (2022): 2398–2405, 10.1038/s41591-022-02051-3. PMC9671810 36357676

[45] A. Shafiee , M. M. Teymouri Athar , M. J. Amini , et al., “Reactivation of Herpesviruses During COVID‐19: A Systematic Review and Meta‐Analysis,” Reviews in Medical Virology 33, no. 3 (2023): e2437, 10.1002/rmv.2437. 36880642

[46] E. Shikova , V. Reshkova , A. Kumanova capital , et al. On Behalf Of The European Network On Me/Cfs, E, “Cytomegalovirus, Epstein‐Barr Virus, and Human Herpesvirus‐6 Infections in Patients With Myalgic Small ie, Cyrillicncephalomyelitis/Chronic Fatigue Syndrome.” Journal of Medical Virology (2020). 92, 12 3682–3688, 10.1002/jmv.25744. 32129496 PMC7687071

[47] M. Ruiz‐Pablos , B. Paiva , R. Montero‐Mateo , N. Garcia , and A. Zabaleta , “Epstein‐Barr Virus and the Origin of Myalgic Encephalomyelitis or Chronic Fatigue Syndrome,” Frontiers in Immunology 12 (2021): 656797, 10.3389/fimmu.2021.656797. 34867935 PMC8634673

[48] M. Patnaik , A. L. Komaroff , E. Conley , E. A. Ojo‐Amaize , and J. B. Peter , “Prevalence of IgM Antibodies to Human Herpesvirus 6 Early Antigen (p41/38) in Patients With Chronic Fatigue Syndrome,” Journal of Infectious Diseases 172, no. 5 (1995): 1364–1367, 10.1093/infdis/172.5.1364. 7594679

[49] A. L. Komaroff , “Is Human herpesvirus‐6 a Trigger for Chronic Fatigue Syndrome?,” Journal of Clinical Virology 37 (2006): S39–S46. 17276367 10.1016/S1386-6532(06)70010-5

[50] A. L. Komaroff and W. I. Lipkin , “Me/Cfs and Long COVID Share Similar Symptoms and Biological Abnormalities: Road Map to the Literature,” Frontiers in Medicine 10 (2023): 1187163, 10.3389/fmed.2023.1187163. 37342500 PMC10278546

[51] M. K. Smatti , F. S. Cyprian , G. K. Nasrallah , A. A. Al Thani , R. O. Almishal , and H. M. Yassine , “Viruses and Autoimmunity: A Review on the Potential Interaction and Molecular Mechanisms,” Viruses 11, no. 8 (2019): 762, 10.3390/v11080762. 31430946 PMC6723519

[52] G. S. Taylor , H. M. Long , J. M. Brooks , A. B. Rickinson , and A. D. Hislop , “The Immunology of Epstein‐Barr Virus‐Induced Disease,” Annual Review of Immunology 33 (2015): 787–821, 10.1146/annurev-immunol-032414-112326. 25706097

[53] T. V. Lanz , R. C. Brewer , P. P. Ho , et al., “Clonally Expanded B Cells in Multiple Sclerosis Bind EBV EBNA1 and GlialCAM,” Nature 603, no. 7900 (2022): 321–327, 10.1038/s41586-022-04432-7. 35073561 PMC9382663

[54] A. Vojdani , E. Vojdani , E. Saidara , and M. Maes , “Persistent SARS‐CoV‐2 Infection, EBV, HHV‐6 and Other Factors May Contribute to Inflammation and Autoimmunity in Long COVID,” Viruses 15, no. 2 (2023): 400, 10.3390/v15020400. 36851614 PMC9967513

[55] D. Verma , T. M. Church , and S. Swaminathan , “Epstein‐Barr Virus Lytic Replication Induces ACE2 Expression and Enhances SARS‐CoV‐2 Pseudotyped Virus Entry in Epithelial Cells,” Journal of Virology 95, no. 13 (2021): e0019221, 10.1128/JVI.00192-21. 33853968 PMC8316011

[56] J. E. Gold , R. A. Okyay , W. E. Licht , and D. J. Hurley , “Investigation of Long Covid Prevalence and Its Relationship to Epstein‐Barr Virus Reactivation,” Pathogens 10, no. 6 (2021): 763, 10.3390/pathogens10060763. 34204243 PMC8233978

[57] S. Zubchenko , I. Kril , O. Nadizhko , O. Matsyura , and V. Chopyak , “Herpesvirus Infections and Post‐COVID‐19 Manifestations: A Pilot Observational Study,” Rheumatology International 42, no. 9 (2022): 1523–1530, 10.1007/s00296-022-05146-9. 35650445 PMC9159383

[58] J. Rohrhofer , M. Graninger , L. Lettenmaier , et al., “Association Between Epstein‐Barr‐Virus Reactivation and Development of Long‐COVID Fatigue,” Allergy 78, no. 1 (2023): 297–299, 10.1111/all.15471. 35950630 PMC9538037

[59] M. J. Peluso , T. M. Deveau , S. E. Munter , et al., “Chronic Viral Coinfections Differentially Affect the Likelihood of Developing Long COVID,” Journal of Clinical Investigation 133, no. 3 (2023): e163669, 10.1172/JCI163669. 36454631 PMC9888380

[60] A. Ali , M. Ohashi , A. Casco , et al., “Rta is the Principal Activator of Epstein‐Barr Virus Epithelial Lytic Transcription,” PLoS Pathogens 18, no. 9 (2022): e1010886, 10.1371/journal.ppat.1010886. 36174106 PMC9553042

[61] R. A. Wood , L. Guthridge , E. Thurmond , et al., “Serologic Markers of Epstein‐Barr Virus Reactivation are Associated With Increased Disease Activity, Inflammation, and Interferon Pathway Activation in Patients With Systemic Lupus Erythematosus,” Journal of Translational Autoimmunity 4 (2021): 100117, 10.1016/j.jtauto.2021.100117. 35005588 PMC8716608

[62] R. Fernandez‐Ruiz and T. B. Niewold , “Type I Interferons in Autoimmunity,” Journal of Investigative Dermatology 142, no. 3 Pt B (2022): 793–803, 10.1016/j.jid.2021.11.031. 35016780 PMC8860872

[63] S. J. Lee , T. Yoon , J. W. Ha , et al., “Prevalence, Clinical Significance, and Persistence of Autoantibodies in Covid‐19,” Virology Journal 20, no. 1 (2023): 236, 10.1186/s12985-023-02191-z. 37845706 PMC10577963

[64] K. Son , R. Jamil , A. Chowdhury , et al., “Circulating Anti‐Nuclear Autoantibodies in COVID‐19 Survivors Predict Long Covid Symptoms,” European Respiratory Journal 61, no. 1 (2022): 2200970, 10.1183/13993003.00970-2022. PMC9515477 36137590

[65] M. Rojas , Y. Rodríguez , Y. Acosta‐Ampudia , et al., “Autoimmunity is a Hallmark of Post‐COVID Syndrome,” Journal of Translational Medicine 20, no. 1 (2022): 129, 10.1186/s12967-022-03328-4. 35296346 PMC8924736

