# Supplementary material for: Seasonal Coronavirus‐Induced Immunological Imprinting and Previous Herpesvirus Infections in Patients With Long COVID
Source: J Med Virol. 2025 Aug 30;97(9):e70582. doi: 10.1002/jmv.70582 (PMC12397718; doi:10.1002/jmv.70582)
Supplement: Supplementary file 1 — Supplementary Table 1: Associatons between anti‐coronavirus antibodies and fatigue and cognitive failure severity in LC patients. Supplementary Figure 1: SARS‐CoV‐2‐specific IgG, IgA, and IgM antibody levels in LC patients infected with the Alpha, Delta, or Omicron variant. Supplementary Figure 2: HKU1 and OC43 spike‐specific antibody levels in LC patients infected with the Alpha, Delta, or Omicron variant. [file JMV-97-e70582-s001.pdf]

Supplementary Table 1: Associations between anti-coronavirus antibodies and fatigue and cognitive failure severity in LC patients. Antibody results from Figure 1 and Figure 2 that were significantly different between LC patients and HC were selected for this analysis. P-values were adjusted for multiple comparisons using the Benjamini-Hochberg method.

	**FAS**		**CFQ**	
	Spearman r	P value	Spearman r	P value
SARS-CoV-2 Spike IgG	-0.231	0.384	-0.085	0.875
SARS-CoV-2 S1 IgG	-0.217	0.384	-0.042	0.875
SARS-CoV-2 N IgG	-0.033	0.824	0.023	0.875
SARS-CoV-2 Spike IgA	-0.344	0.194	-0.069	0.875
SARS-CoV-2 S1 IgA	-0.177	0.384	-0.102	0.875
SARS-CoV-2 N IgA	-0.201	0.384	-0.249	0.335
SARS-CoV-2 Spike IgG/IgM ratio	-0.160	0.386	-0.154	0.829
SARS-CoV-2 S1 IgG/IgM ratio	-0.234	0.384	-0.114	0.875
HKU1 Spike IgG	-0.173	0.384	-0.344	0.108
OC43 Spike IgG	-0.139	0.429	-0.339	0.108
OC43 S2 IgA	-0.115	0.487	0.035	0.875

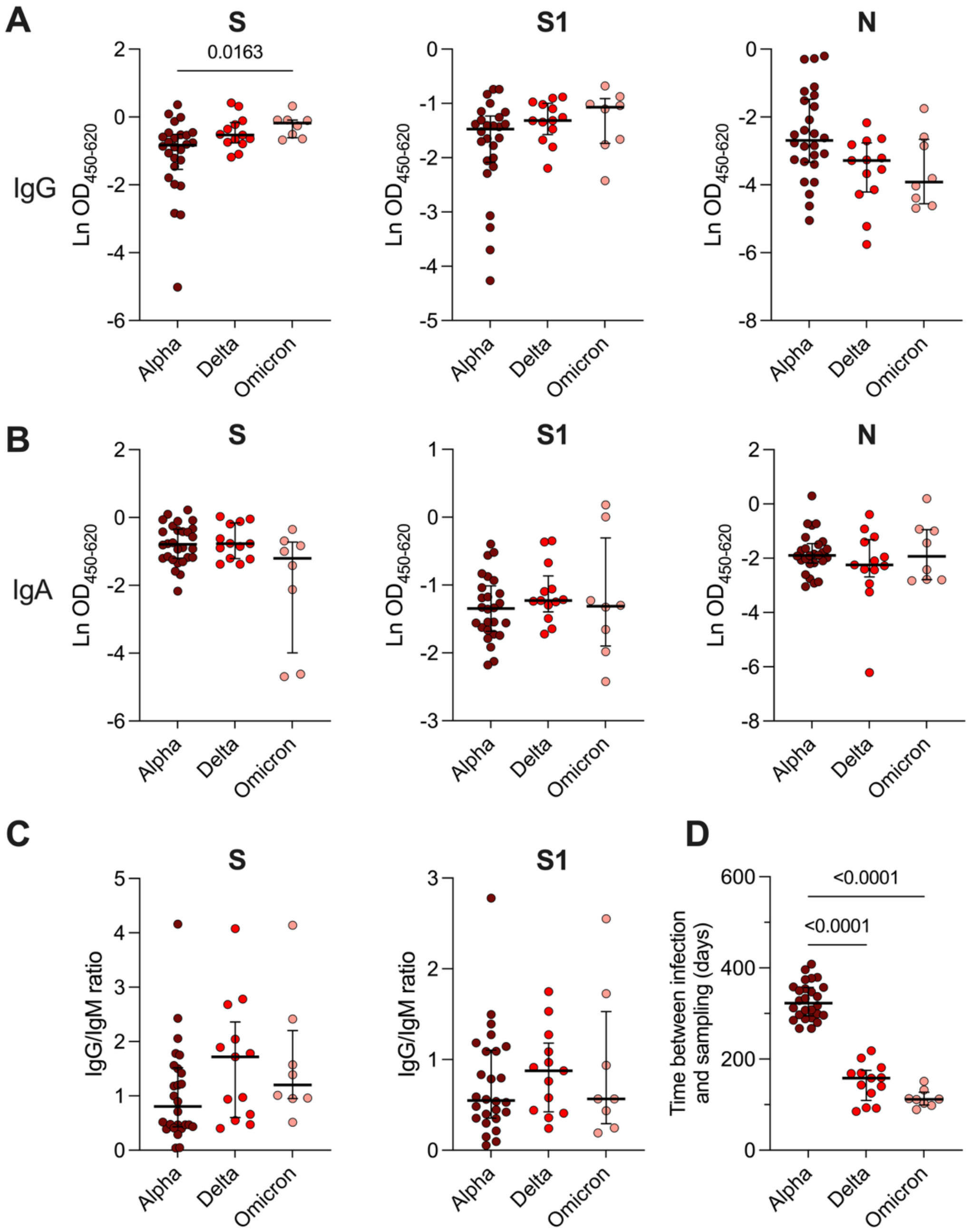

Supplementary Figure 1. SARS-CoV-2-specific IgG, IgA, and IgM antibody levels in LC patients infected with the Alpha, Delta, or Omicron variant. (A) IgG and (B) IgA antibody levels against the full spike (S) protein, spike subunit 1 (S1), and nucleocapsid (N) protein of SARS-CoV-2. (C) SARS-CoV-2 S and S1 IgG/IgM ratios. OD450-620 values were used for ratio calculations. (D) Time between infection and blood collection for LC patients infected with the Alpha, Delta, or Omicron variant. Data are presented as median with IQR and statistical comparisons were made using the Kruskal-Wallis test with Dunn's post hoc analysis.

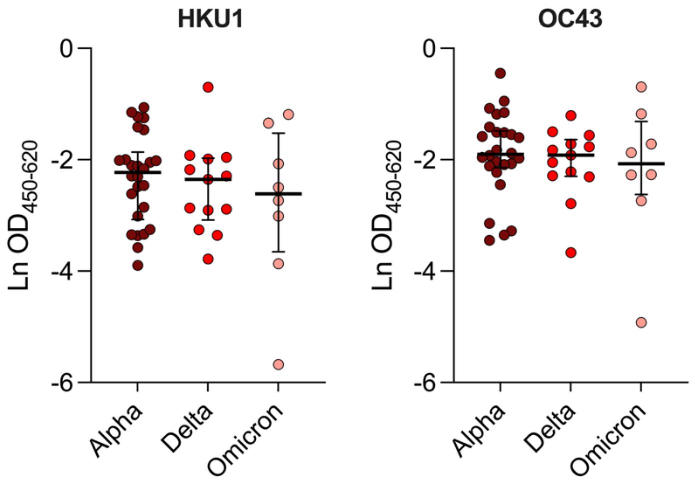

Supplementary Figure 2. HKU1 and OC43 spike-specific antibody levels in LC patients infected with the Alpha, Delta, or Omicron variant. Data are presented as median with IQR and statistical comparisons were made using the Kruskal-Wallis test with Dunn's post hoc analysis.
